# Emerging Role of Nuclear Medicine in Prostate Cancer: Current State and Future Perspectives

**DOI:** 10.3390/cancers15194746

**Published:** 2023-09-27

**Authors:** Fabio Volpe, Carmela Nappi, Leandra Piscopo, Emilia Zampella, Ciro Gabriele Mainolfi, Andrea Ponsiglione, Massimo Imbriaco, Alberto Cuocolo, Michele Klain

**Affiliations:** Department of Advanced Biomedical Sciences, University of Naples Federico II, 80138 Naples, Italy; fabio.volpe@unina.it (F.V.); c.nappi@unina.it (C.N.); lea-17-08@hotmail.it (L.P.); emilia.zampella@gmail.com (E.Z.); c.mainolfi@libero.it (C.G.M.); a.ponsiglionemd@gmail.com (A.P.); mimbriaco@hotmail.com (M.I.); cuocolo@unina.it (A.C.)

**Keywords:** prostate cancer, theragnostic, nuclear medicine, targeted therapy, radioligand therapy, PET/CT

## Abstract

**Simple Summary:**

The huge armamentarium of currently available theragnostic modalities allows a novel approach to prostate cancer from imaging to therapy. Clinical examination is the starting-point, then radiology and nuclear medicine are often needed to define the illness grading to set up the best therapeutic strategy. Prostate cancer care horizons are opening with the aid of nuclear medicine, which takes advantage of the technological ascendancy of prostate-specific membrane antigen-based imaging and therapy and is currently evolving with machine-learning approaches. We have focused our review on the current state, on the advancements, and on the future prospects of nuclear medicine modalities that could change prostate cancer’s standard of care.

**Abstract:**

Prostate cancer is the most frequent epithelial neoplasia after skin cancer in men starting from 50 years and prostate-specific antigen (PSA) dosage can be used as an early screening tool. Prostate cancer imaging includes several radiological modalities, ranging from ultrasonography, computed tomography (CT), and magnetic resonance to nuclear medicine hybrid techniques such as single-photon emission computed tomography (SPECT)/CT and positron emission tomography (PET)/CT. Innovation in radiopharmaceutical compounds has introduced specific tracers with diagnostic and therapeutic indications, opening the horizons to targeted and very effective clinical care for patients with prostate cancer. The aim of the present review is to illustrate the current knowledge and future perspectives of nuclear medicine, including stand-alone diagnostic techniques and theragnostic approaches, in the clinical management of patients with prostate cancer from initial staging to advanced disease.

## 1. Introduction

**Figure 1:**
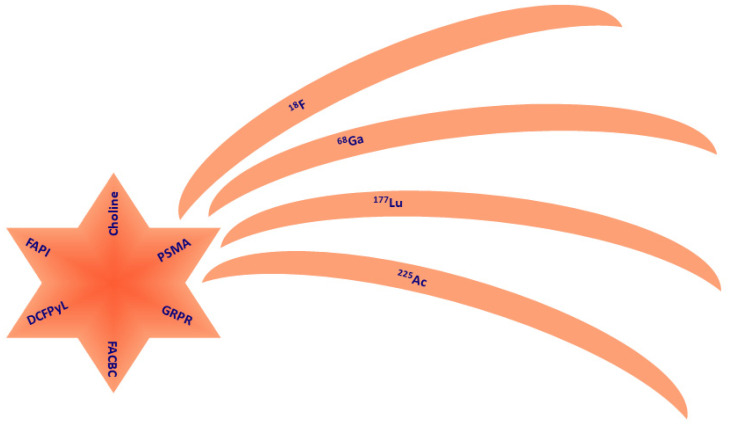
Pictorial scheme of the available ligands (comet nucleus) and isotopes (comet tails) with application to prostate cancer management. PSMA is the only theragnostic compound.

Prostate cancer (PC) is the most frequent epithelial neoplasia after skin cancer in men starting from 50 years [1], but it is often not clinically evident at the early stage as prostatic intraepithelial neoplasia [2]. Not every intraepithelial neoplasia can progress to PC, even in high-grade cases. Prostate-specific antigen (PSA) dosage can be used as an early diagnostic tool, starting from the age of 50 years when the risk of PC increases. In the presence of specific risk factors such as family history, African ethnicity and BRCA1/2 carriers, screening should be brought forward to 45 years [3]. The standard approach to PC diagnosis comprises PSA screening and digital rectal examination. Multiparametric magnetic resonance (MR) and eventual prostate biopsy may be second-level tests [4,5,6]. Not every PC needs to be treated immediately, especially at early presentation, and it can be considered a chronic disease [7,8]. However, risk stratification (from very low to high risk) is the mainstream contention of modern guidelines to evaluate treatment options in more aggressive disease [8,9]. Different treatment options, ranging from early aggressive treatments, such as radical prostatectomy and radical radiotherapy, to deferred treatments (i.e., treating men when the disease progresses and becomes symptomatic), depend on parameters such as tumor grade and tumor stage. Imaging plays an important role both in the non-invasive detection, localization, grading, and staging of PC and in guiding histopathologic analysis by biopsies. In particular, magnetic resonance (MR) has become a powerful tool for achieving these goals [10,11,12,13,14,15]. Furthermore, the recent introduction of specific prostate ligands that can carry radioactive isotopes including β+ (^18^F, ^68^Ga), β-/γ(^177^Lu), and α (^225^Ac) emitters with diagnostic and therapeutic characteristics (Figure 1) may redefine the role of nuclear medicine in PC [16].

The aim of the present review is to illustrate current knowledge and future perspectives of nuclear medicine, including stand-alone diagnostic techniques and theragnostic approaches, in the clinical management of patients with prostate cancer from initial staging to advanced disease.

## 2. Staging Prostate Cancer

The need for a reproducible description of cancer spread has been met by the creation of the staging system. The American Joint Committee on Cancer’s TNM system is widely used, but the most recent update dates from 2018 [17]. The TNM system for PC is based on the PSA level at the time of diagnosis; the extent of the primary tumor is described by the T parameter, the involvement of lymph nodes is described by the N parameter, and cancer extension to other regions of the body is described by the M parameter. However, the National Comprehensive Cancer Network (NCCN) Guidelines Version 1.2023 for Prostate Cancer treatment recommendations, released recently [18], are based on a risk stratification that includes TNM staging rather than on the American Joint Committee on Cancer prognostic grouping. The grading system for PC (based on the Gleason score) tries to measure how likely it is that the cancer will grow and spread quickly. This is determined by the results of the direct prostate examination by the pathologist.

However, the prognostic value of American Joint Committee on Cancer 8th edition staging is not applicable to some staged patients. Higher PSA levels or higher tumor grade are associated with a worse prognosis than that of patients with a higher stage but lower PSA level or lower tumor grade [19]. Several studies also showed that the high-grade group has a significantly worse prognosis than the lower-grade group and should be considered a distinct group [20,21,22,23]. Early-stage patients are commonly diagnosed with a localized, low-risk PC with excellent treatment outcomes [24,25,26]. Along with PSA, some other markers have been proposed. For instance, PC gene 3, an overexpressed long non-coding RNA, is detectable in urine sediments [27,28,29]. This gene correlates with cancer volume, but some data cast doubt on its correlation with grade [30]. A PC gene 3 test is now used to settle a second biopsy after a negative one [31]. The first attempt at risk categorization was made by D’Amico and co-workers that classified patients into low-, intermediate-, and high-risk groups according to PSA, tumor stage, and Gleason score at the moment of diagnosis [32]. Clinical therapeutic and technological upgrades have stimulated the need for a more precise risk assessment, because of the availability of a large variability of therapeutic choices and of a cost-effective option. The National Institute for Health and Care Excellence, European Association of Urology, Genito-Urinary Radiation Oncologists of Canada, American Urological Association, National Comprehensive Cancer Network, and Cambridge Prognostic Groups risk group systems have been created, which then evolved into the Cancer of the Prostate Risk Assessment score [33] and the Memorial Sloan Kettering Cancer Center nomogram [34]. Nomograms attempt to address the large amount of single risk factors and imaging features that may affect the prognosis and the therapeutic strategy of PC patients, with a simple readability of responses [35].

## 3. Radiological Imaging

### 3.1. Ultrasound

Following an abnormal PSA level or digital rectal examination, transrectal ultrasonography (TRUS) is frequently used as the first step in the diagnostic process to identify abnormalities and direct biopsies. Because of the high frequency of multiple localization, systematic sextant biopsies are indicated [36]. On ultrasonography, the primary PC can be hyper-echoic or isoechoic (30–40% of lesions), although it is typically detected as a hypoechoic lesion (60–70%) at the gland’s periphery [37]. The preferred technique for implanting brachytherapy seeds in the prostate is transrectal ultrasonography [38].

### 3.2. Magnetic Resonance

**Figure 2:**
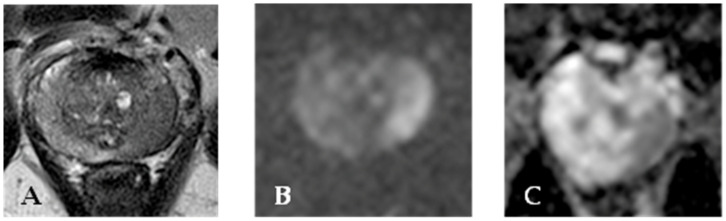
MR of a peripheral left lobe PC. T2 weighted sequence (**A**) shows an area of hyperintensity in the left lobe. This finding is confirmed on apparent diffusion coefficient sequences (**B**). DWI sequence (**C**) reveals hyperintensity in the lesion area.

After ultrasound-guided prostate biopsy, MR plays a major role in the evaluation of known PC in order to determine if there is extracapsular extension [39,40,41]. Thus, MR can detect and localize cancer when the PSA is constantly elevated, but routine TRUS biopsy is negative. Both the American College of Radiology and the European Society of Uroradiology encourage the implementation of multiparametric MR for PC assessment consisting in a combination of T2-weighted imaging with functional techniques such as diffusion-weighted imaging, dynamic contrast-enhanced imaging, and spectroscopy [12]. MR can guide prostate biopsy, in the case of negative TRUS biopsy but high clinical suspicion due to elevated PSA levels [42,43]. MR has also has a role in PC patients surgically treated with radical prostatectomy. The use of multi-parametric MR is helpful in detecting and localizing a prostatic lesion [44]. T1 signal used as morphological examination can define prostate contour, neurovascular bundle incasement, and post-biopsy bleeding [45]. T2-weighted images acquired with an endorectal coil show PC usually appearing as a low-signal area within a normally high-signal peripheral zone [46,47]. Diffusion-weighted imaging (DWI) is the crucial sequence for peripheral zone tumor detection [48]. DWI/apparent diffusion coefficient sequences demonstrate restricted diffusion. Dynamic contrast imaging shows enhancement, but it is often a challenge in the central zone to distinguish lesions from prostatitis or benign prostatic hyperplasia. In addition, it has more specificity than T2 signal but extends the post-processing time [49,50]. MR findings can also be expressed by the Prostate Imaging Reporting and Data System (PI-RADS) score. PI-RADS has been released by a consensus of American College of Radiology, European Society of Urogenital Radiology, and AdMeTech Foundation experts to address and homologate the diagnosis likelihood of clinically significant cancer from MR findings. The latest revision (PI-RADS 2.1) was released in 2019 [51,52] and its clinical use is supported by the literature [53], but the necessity of a more precise assessment has led to the introduction of nomograms to integrate clinical, biological, and imaging data to improve diagnosis performance [54]. PI-RADS scoring has a good correlation with malignant prostate findings and thus with the Gleason score. This latter recognizes a primary and a secondary pattern, as well as five sub-patterns in each. The sum of the two patterns provides the Gleason score, which has prognostic significance. Patients with a low Gleason score do well clinically, whereas patients with a high score do poorly. Figure 2 provides a representative example of MR findings in a PC patient.

### 3.3. Magnetic Resonance Spectroscopy

Some metabolites such as choline citrate or choline creatine are increased in PC cells [55] and their levels can be evaluated by MR spectroscopy. The multiparametric techniques have been increasingly used in the assessment of prostate malignancy with MR but some issues have emerged with the use of powerful magnets, such as radiofrequency field dis-homogeneity and high local specific absorption rates, that may increase local heating into the body tissues and give rise to safety concerns [56,57]. While T1-weighted images can better describe lymphadenopathy, MR spectroscopy associated with fast T2-weighted imaging is a promising technology for the detection of primary disease [57]. The prostate physiologically produces citrate from the peripheral zone while PC cells do not [58]. Thus, citrate and polyamine levels are high and choline levels low in normal prostatic tissue while they have inverted concentrations in PC [59]. MR spectroscopy is definitely a powerful instrument and can be a game changer in border-line patients [60], but a standardization of findings is needed as inter-operator variability can affect medical report repeatability.

### 3.4. Computed Tomograhy

CT of the abdomen and pelvis and whole-body bone scans remain the standard of care for the detection of visceral, nodal, and bone metastasis. CT is not perfect at detecting in situ PC, and abdomen and pelvis scans are commonly used to finalize radiation therapy planning. In advanced PC, a CT is used for staging purpose, to detect metastatic lymph nodes in pelvis and the retroperitoneal space, hydronephrosis, and osteoblastic metastases [61].

## 4. Nuclear Medicine Imaging

### 4.1. Planar Scintigraphy and Single-Photon Emission Computed Tomography

Bone scintigraphy with planar and single-photon emission computed tomography (SPECT) imaging can detect high uptake of ^99m^Tc-methylenediphosphonate (^99m^Tc-MDP) as result of the bone metabolism. It is not a tumor-specific tracer; in fact, its uptake is higher at bone repair loci where bone metastasis can be located. PC bone metastases are osteoblastic; hence a bone scan can detect them as hot spots or localized accumulation [62]. Currently, bone scintigraphy is one of the first-line imaging techniques for staging and follow-up of PC bone metastasis, but the classical approach is only qualitative with low specificity. Some software-based indexes have been proposed to optimize bone scintigraphy medical reports [63,64,65,66]. The recent introduction of prostate-specific membrane antigen (PSMA) ligands has the potential to rapidly supersede bone scans with ^99m^Tc-MDP [67] when they have been demonstrated to be a cost-efficient imaging modality.

### 4.2. Choline

**Figure 3:**
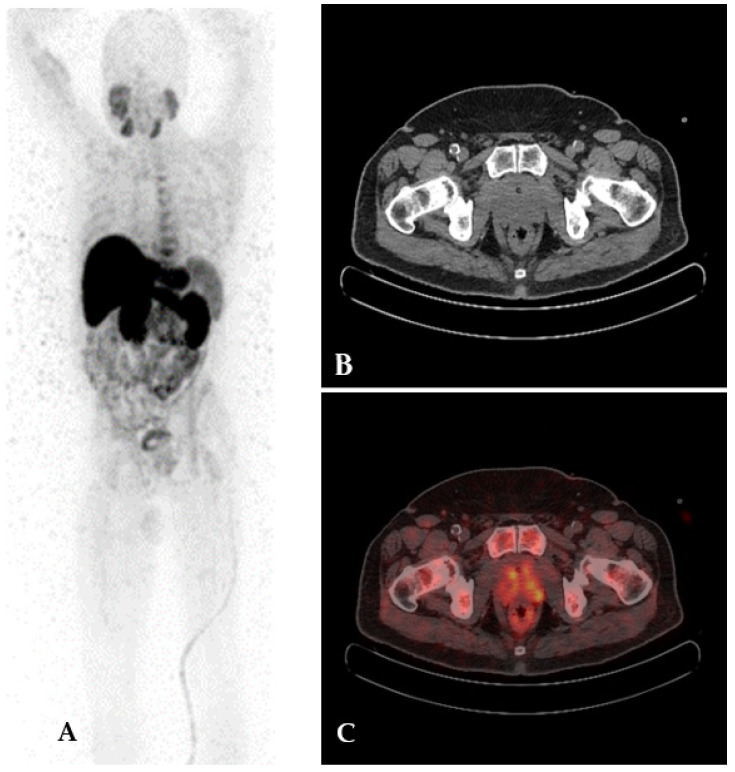
^18^F-choline PET/CT imaging in a patient with prostate cancer. Maximum intensity projection (**A**), CT (**B**), and fused (**C**) axial slices show increased tracer uptake in the upper lobes of the prostate.

**Figure 4:**
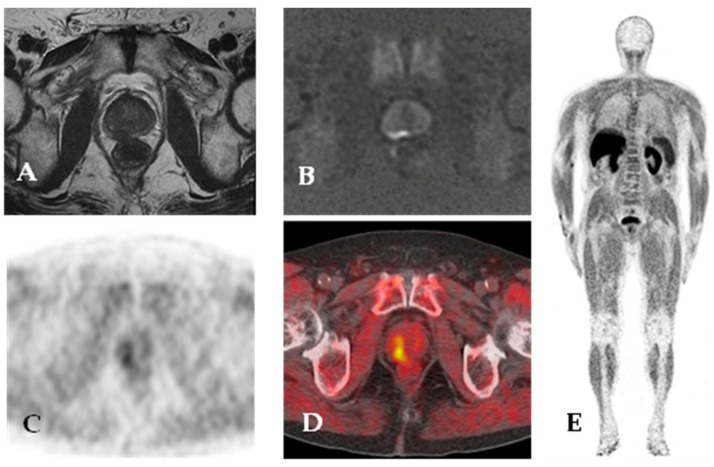
^18^F-choline PET/MR. Staging MR imaging shows a large peripheral zone (right posterior mid-gland) lesion with low signal on T2-weighted images (**A**) and focal and marked hyperintensity on high b value DWI (**B**). ^18^F-choline PET/CT images (**C**,**D**) show increased tracer uptake in the right posterior area of the prostate gland. Maximum intensity projection (**E**) ^18^F-choline PET images.

In the 90s, choline was proposed as a positron emission tomography (PET) radiotracer for tumor detection with low urinary excretion [68]. Tumor cells need choline to make phosphatidylcholine and other choline-derived membrane constituents. Biochemical analyses have demonstrated that choline kinase activity is increased in tumor cells [69,70]. ^18^F-choline PET/CT can be a good tool in PC patients at a high risk of extracapsular disease and before surgery to exclude distant metastases [71] but it has demonstrated low sensitivity, despite good specificity, in the evaluation of nodal localization [72]. ^18^F-choline PET/CT is also characterized by high detection rate of local and distant recurrence post initial treatment of PC [73,74]. ^11^C-choline is a valuable radiotracer, especially in bone metastasis assessment. ^11^C has a higher positron energy than ^18^F (390 vs. 252 MeV) and longer positron range (1.27 vs. 0.66 mm), which is in theory a disadvantage for image quality [75], but probably can be useful in a dense stroma like bone. ^11^C-choline PET/CT PC detection performance varies, as reported in numerous studies [76,77,78,79]. This is probably due to the heterogeneity of patient samples regarding PSA level, staging, and castration therapy. As proposed by some authors, PSA measurement is involved in the detection rate of PC recurrence by ^11^C-choline PET/CT [76,80,81]; in particular, PSA doubling time and PSA velocity are predictors for pathological PET scan findings. Figure 3 and Figure 4 illustrate ^18^F-choline PET/CT and PET/MR imaging findings in patients with PC.

### 4.3. Prostate-Specific Membrane Antigen

**Figure 5:**
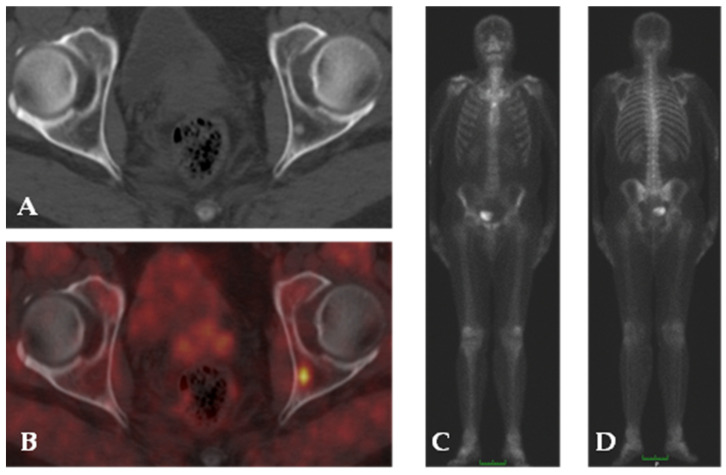
^68^Ga-PSMA PET/CT imaging in a patient with PC. Co-registered CT (**A**) shows hyper-dense area in the left iliac bone with no evidence of focal uptake of ^68^Ga-PSMA on fused images (**B**). ^99m^Tc-MDP bone scan anterior and posterior projection (**C**,**D**) shows normal tracer distribution.

PSMA is a type II membrane glycoprotein that activates the protein kinase B pathway [82]. It has been proposed as PC marker because it is expressed at very high levels in PC cells but at lower levels also in normal prostate tissue, in the peripheral and central nervous system, in the bowel, and in the salivary glands [83]. Nevertheless, PSMA imaging has not demonstrated uptake in spinal cords without PC localization, and thanks to the blood–brain barrier, PSMA brain uptake has not been seen [84]. PSMA expression in the gastrointestinal tract is responsible for hydrolysis of poly-glutamate folates contained in foods. Folate is then transported into enterocytes and then to the liver [85]. PSMA is also known as folate hydrolase 1 and is involved in folate uptake; its substratum, however, can change in different tissues, for instance, malignant neo-vasculature [85,86,87]. The role of PSMA in carcinogenesis has been widely debated. Probably its overexpression can alter the G2/M cell cycle, eliciting aneuploidy [88]. Folates provide activation of the protein kinase B pathway in vitro and PSMA overexpression has been observed in more aggressive PC [82]. Given high PSMA expression in aggressive PC, patients probably must be careful about dietary habits [89]. The evolution of theragnostics from the first released cancer-specific antibodies to current tracers that can heal cancer with targeted radionuclide administration sparing normal tissues can be considered exceptional. Considering PSMA as a target for theragnostic purposes has been a natural consequence. ^111^In-capromab pendetide, a mouse monoclonal antibody marked with ^111^In, was the first tracer for PSMA SPECT imaging in PC patients; however, it was not perfect [90]. PSMA targeting has evolved with the introduction of a group of low-molecular-weight ligands (MIP-1095, MIP-1404, PSMA-11/617/1007, Piflufolastat, PSMA I&T) marked with ^18^F and ^131^I for imaging and therapy purposes, respectively, and has been studied in several US trials. In particular, PSMA can be labelled with ^68^Ga or ^18^F for PET/CT imaging. While ^18^F has better physics characteristics than ^68^Ga due to its lower positron range (2 mm vs. 3.5 mm), ^68^Ga and ^18^F have demonstrated similar performance in malignancy detection [91]. Nevertheless, ^18^F-PSMA seems to be sightly linked to potential false-positive findings, especially in bone tissue [92,93]. The role of PSMA-targeted imaging in initial staging and re-staging of PC has been proven as an effective option. CT, MR, and bone scans lack sensitivity and specificity for detecting occult metastatic disease, in particular in the case of low PSA values [11]. When compared to radio-labelled choline, PSMA imaging shows its strength in early PC recurrence detection, with PSA blood dosage lower than 1 ng/mL [94,95]. In addition, PSMA PET/CT imaging can better estimate PC tumor burden [96]. Figure 5 demonstrates the clear superiority of ^68^Ga-PSMA PET/CT over bone scan scintigraphy with ^99m^Tc-MDP. Recently, the European Association of Nuclear Medicine and the Society of Nuclear Medicine and Molecular Imaging published the second release of their procedure guidelines for PC imaging [97]. This joint venture updated the specific PET/CT indications for PSMA ligands that include the initial staging of intermediate–high risk PC, the localization of metastasis in biochemical recurrent or persistent PC, especially when other imaging methods have failed, and staging and re-staging prior to and after radioligand therapy of PC [97]. This statement is confirmed by several PC-specific guidelines such as those of the European Association of Urology, European Society for Medical Oncology, National Comprehensive Cancer Network; and American Society of Clinical Oncology [3,15,38,98]. Their high sensitivity and specificity can be exploited by further improving MR-guided biopsy with multimodal imaging offered by PET/MR, as proposed by the study by Ferraro and coworkers [99]. PSMA ligand PET/TC needs some education in interpretation of uptake foci. Inflammation and infection, salivary glands, ganglia (stellate and celiac), gall bladder, and all kinds of prostate pathology can be associated with an increased PSMA uptake, whereby it can mimic a malignancy localization [100]. To address this issue, PSMA-RADS Version 1.0 [101] and Prostate Cancer Molecular Imaging Standardized Evaluation (PROMISE) criteria have been developed and are currently evolving with clinical experience and technological advancement [102].

### 4.4. Piflufolastat

Among PSMA ligands boasting high affinity for their extracellular domain, piflufolastat labeled with ^18^F (^18^F-DCFPyL) has been extensively investigated, demonstrating superior performance for the staging and re-staging of PC over traditional imaging modalities in the OSPREY and CONDOR clinical trials [103,104]. Szabo and coworkers [105] were the first to prospectively evaluate ^18^F-DCFPyL in nine hormone-naïve and castration-resistant PC (crPC) patients with metastatic evolution confirmed by histological examination. Dosimetric evaluation revealed that the kidneys adsorbed the highest dose, followed by the bladder wall, submandibular glands, and liver, with a distribution pattern similar to ^18^F-FDG, while physiological bio-distribution was seen in the liver, spleen, kidneys, the lacrimal and salivary glands, and the small bowel. Jansen et al. [106] reported high repeatability of both lesion detection rate and uptake in 12 patients with PC. Parameters such as volume and standardized uptake values showed better accuracy with ^18^F-DCFPyL, particularly for lymph node localization. If changes in semi-quantitative parameters are recorded between baseline and follow-up ^18^F-DCFPyL PET/CT, the reader is confident that such findings are not caused by uptake variability, suggesting that this compound may be a reliable image biomarker for response assessment [107]. Li et al. [108] also reported on variability in normal organ uptake using ^18^F-DCFPyL and demonstrated less variability in normal liver relative to other organs. Of note, the variability was even lower when compared to liver uptake using ^18^F-FDG (coefficient of variation, ^18^F-DCFPyL, 13.8–14.5% vs. ^18^F-FDG, 21–23%) [108,109]. In addition, a recent study investigated whether uptake in normal organs correlates with higher tumor burden [106]. Of note, in patients with high ^18^F-DCFPyL uptake metastatic volume, the tumor sink effect was minimal. However, it should be considered that inter-patient and intra-patient factors may impact the intrinsic organ variability [110]. However, dosimetry for PSMA-targeted radioligand therapy could be further improved and PET protocols could be better refined to enhance tracer uptake in putative sites of disease. A recent randomized phase 2 research clinical trial (ORIOLE) underlined the potential role of PSMA-targeted radioligand PET in directing and enhancing the therapeutic efficiency of metastatic-directed therapy administered by radionuclide therapy, and demonstrated that individuals with total consolidation of disease detectable by PSMA-targeted PET-CT were associated with lower risk of new metastases at 6 months [111]. This work serves as an excellent example of the crucial function that PSMA imaging plays.

### 4.5. Fluciclovine

^18^F-fluciclovine is an amino-acid analogue that takes advantage of the increased energy demand of PC and acts as a radiotracer when labeled with ^18^F. It is taken in to the cells by facilitated transport, in particular by alanine-serine-cysteine transporter and L-type amino acid 1 transporter [112,113,114]. The US Food and Drug Administration and the European Medicines Agency approved ^18^F-fluciclovine as a PET radiotracer in men with PC with biochemical recurrence after radical prostatectomy or radiotherapy [113]. Uptake is normally high in the liver and pancreas, while the salivary glands, pituitary, lymphoid tissue of Waldeyer’s ring, thyroid, breast parenchyma, esophagus, stomach, adrenal glands, bowel, and renal parenchyma show lower distribution. Although it has been shown that urinary excretion is low, and it offers high contrast for primary PC detection, some studies have demonstrated that ^18^F-fluciclovine PET cannot independently characterize primary lesions requiring integration with multiparametric MR findings [115,116], so ^18^F-fluciclovine cannot be used for PC staging. PC recurrence identification, especially in the pelvis, is the most promising field of use of ^18^F-fluciclovine in the near future [117].The role of ^18^F-fluciclovine PET/CT in PC patients with biochemical recurrence after curative-intent primary therapy has been studied by the LOCATE (NCT02680041) [118] and FALCON (NCT02578940) [119] trials. They found that this tracer could lead to the most appropriate treatment approach by determining the tumor burden and location linked to biochemical recurrence. In addition,^18^F-fluciclovine has demonstrated a superior recurrence detection performance over ^18^F-choline PET/CT, especially in patients with blood PSA level inferior to 1 ng/mL [120,121,122,123]. So, this radiolabeled compound can be considered to assess earlier PC recurrency, with a possible advantage in clinical management and prognosis. However, ^18^F-choline PET/CT performance does not seems to be superior to the more accessible radiolabeled PSMA PET/CT in recurrence detection far from the bladder [124,125].

### 4.6. Fluorodeoxyglucose

^18^F-fluorodeoxyglucose (FDG) is the first radiotracer utilized for human brain PET and still is the radiotracer of choice in several PET/CT oncologic applications. ^18^F-FDG is rapidly captured from plasma by cells, and then the ^18^F-FDG takes advantage of the Warburg effect and is phosphorylated to prevent further metabolism and blood recirculation [126]. ^18^F-FDG is excreted by the kidneys, concentrated in urine and the bladder. Prostate proximity to the bladder limits the utility of ^18^F-FDG PET/TC in primary PC definition [127]. It should be also be taken into consideration that bone scan scintigraphy can be superior to ^18^F-FDG PET/TC for the definition of bone metastasis [128,129,130]. A potential role of ^18^F-FDG PET/TC is as a technique of evaluating mismatches in ^18^F-FDG and prostate-specific tracers PET/TC as an index of cancer de-differentiation with negative prognostic connotation [131,132]. In particular, when PC has become castration-resistant, PSA cannot be considered the best indicator of response to therapy, due to the increase in cancer cell heterogeneity with the presence of PSA-producing and nonproducing cells and the presence of flare phenomena as a result of therapy response. When PC progresses, despite therapy, PSMA uptake may decrease along with an increase in ^18^F-FDG uptake, according to the “flip flop” phenomenon [133]. In this scenario, ^18^F-FDG can be used parallel to PSMA PET/TC and therapy plans can be changed according to PET findings, in order to monitor and treat tumor de-differentiation, if possible [134].

### 4.7. Hetero-Bivalent Agents Targeting Gastrin-Releasing Peptide Receptor or Fibroblast Activation Protein Inhibitor and PSMA

Gastrin-releasing peptide receptor (GRPR) belongs to the bombesin family, and its concentration is highest in the pancreas. Low levels of GRPR are expressed in the bowel and, interestingly, in benign prostate tissue [135]. GRPR is involved in intestinal smooth muscle contraction and increases cancer cell proliferation [136,137]. Over-expression has been observed in PC, but also in breast, lung, head and neck, pancreatic cancers, and malignant brain tumors [138,139]. Radiopharmaceutical engineering has investigated ligands that can target either PSMA or GRPR to improve PC theragnostics. Clinical application is linked to single target expression homogeneity. Heterogeneity of expression of the two targets has been explored by studies of the binding of ^68^Ga-Ga-RM2 and ^68^Ga-Ga-PSMA-11 to their ligands in biochemically recurrent PC [140]. Large areas of negative PSMA expression can be found in primary tumors or metastases, independently of the Gleason score, histological class, and metastasis sites [141]. So, targeting both GRPR and PSMA could be an advantage. On the other hand, PET/CT imaging with radiolabeled fibroblast activation protein (FAP) inhibitor has been proposed in various diseases, including PC. This protein is a serine peptidase expressed in the cell membrane that can be upregulated in activated fibroblasts at wounds, inflammatory sites, and in cancer tissue. Quinoline-based PET tracers acting as FAP inhibitors can accurately detect cancer-activated fibroblasts, demonstrating clear tumor imaging when labeled with ^68^Ga [142,143,144,145].

Table 1 summarizes the available tracers for PET/TC imaging in patients with PC.

## 5. Radioactive Therapy of PC Bone Metastasis

PC can metastasize to bones, especially in advanced stages. Several targeted bone radioactive isotopes, such as ^186^Re, ^89^Sr, and ^153^Sm, were firstly introduced into clinical use for treatment of bone metastasis; then they were superseded by ^223^Ra, an α-emitter, and reserved for palliation therapy in selected patients. The ALSYMPCA trial [113,114,146] was a phase 3 trial that demonstrated that ^223^Ra, an α-emitter, can improve crPC patients’ overall survival compared with placebo and it is well-tolerated. The National Comprehensive Cancer Network also showed first-line use of ^223^Ra for symptomatic bone PC metastasis or bone-predominant disease after chemotherapy in the case of absence of visceral metastases [147]. Bone scintigraphy and ^18^F-fluorocholine PET/CT can help physicians to predict ^223^Ra treatment response. A moderate burden of disease is a good predictor of good response to ^223^Ra [148]. Nevertheless, ^223^Ra has inherent limits because of its restriction to metastatic bone disease uptake. The therapeutic performance of ^223^Ra treatment has been proved by several studies that demonstrate an increase in overall survival and quality of life, along with an excellent safety profile, especially when introduced earlier into the treatment iteration [149,150,151].

## 6. Radioligand Therapy of Advanced Prostate Cancer

The main actors on the ground for a promising novel approach to treat PC are ^177^Lu and ^225^Ac [152]. ^177^Lu is a widely used radioisotope for targeted cancer radiotherapy with a half-life of 6.7 days, emitting both beta particles with an energy of 490 KeV and a therapeutic range of 0.7 to 2.1 mm and gamma rays (112.9 and 208.4 KeV peaks) [153,154,155,156]. ^225^Ac is an alpha emitter (energies ranging from 5.8 to 8.4 MeV) with a half-life of 9.9 days and a very short tissue range (47 to 85 µm) [157]. ^225^Ac can be considered the natural alpha-emitting counterpart to ^177^Lu because of its similar radiolabeling characteristics. Alpha emitters have a clear advantage over beta emitters in cancer cell destruction. They can provoke very efficient double-strand DNA damage, cell cycle arrest; and micronuclei formation, leading ultimately to cell death. Current research suggests that treating the primary tumor or metastases with radionuclides may improve survival in carefully chosen patients with low-volume metastatic PC [158]. However, visceral localization and high alkaline phosphatase are negative predictors of response to ^177^Lu ligand therapy. On the other hand, the onset of high-grade hematotoxicity (anemia, thrombocytopenia, and leukopenia) should be taken into account [159,160]. Although ^177^Lu-PSMA treatment produced an impressive response for pre-treated patients, 40% of PC patients did not respond [161]. Kratochwil et al. [161] tested ^225^Ac-PSMA therapy in strongly pre-treated crPC patients and realized that α-emitters can be more effective than β-emitters. Moreover, α-emitters can have an edge over β-emitters in the case of bone diffused disease, acting as a super-scan for nuclear medicine imaging and sparing bone marrow thanks to low tissue penetration. The VISION phase 3 trial of targeted radioligand therapy including 831 patients demonstrated that radionuclide therapy with ^177^Lu-PSMA extended imaging-based progression-free survival (8.7 vs. 3.4 months) and overall survival (15.3 vs. 11.3 months) when added to standard care in patients with metastatic crPC [162]. TheraP, a phase 2 study, compared ^177^Lu-PSMA to chemotherapy in two hundred men [163]. Prostate-specific antigen (PSA) levels, which usually increase with cancer growth, were monitored. ^177^Lu-PSMA treatment made PSA levels fall by half and, according to the VISION trial, a longer delay of cancer progression was found, confirmed by conventional imaging. ^177^Lu-PSMA was generally well-tolerated, but it also had side effects including fatigue, nausea, kidney complications, and bone marrow suppression. The safety, kinetics, and dosimetry of ^177^Lu-PSMA have been evaluated on a large cohort of patients, demonstrating favorable safety in metastatic crPC patients [164]. The highest absorbed doses among healthy organs were observed for the lacrimal and parotid glands, still not resulting in any significant clinical side effects. Given the encouraging clinical trial results, the European Association of Nuclear Medicine guidelines stated that ^177^Lu-PSMA therapy can be administered when chemotherapy or new androgen-axis compounds therapies have failed, in metastatic crPC with documented PSMA uptake, still with therapeutic purposes rather than with the aim of palliation [165]. However, the safety and efficacy of ^177^Lu-PSMA have suggested potential earlier use in PC [166,167]. Some studies have suggested the possibility of early adoption of ^177^Lu-PSMA therapy, taking advantage of better initial conditions of crPC patients, naïve to chemotherapy, or prior to surgery. [168,169]. However, resistance can occur sometimes. In the LuPIN trial [170], the combination of ^177^Lu-PSMA with NOX66, a flavonoid derivate that activates the mitochondrial caspase system, has been evaluated in end-stage patients, while a ^177^Lu-PSMA combination with abiraterone has been tested in a different sub-trial [171]. ^177^Lu-PSMA can prolong PC patients’ survival, but cannot always inhibit progression, usually to bone but also to liver [155]. In the AlphaBet trial, ^223^Ra has been combined with ^177^Lu-PSMA in order to treat pre-clinical bone micrometastasis and possibly prevent progression to bone, also taking into account that low bone marrow irradiation is a challenge [172]. Drug combinations can be considered as salvage therapies when a single drug fails, but a multimodal approach can also be used. External beam radiation and ^177^Lu-PSMA combined therapy can be considered as an option, but criticality may emerge from the dosimetric point of view [173]. ^177^Lu-PSMA therapy is a reliable option for men with metastatic crPC, but should also have utility in less advanced PC. For now, ^177^Lu-PSMA and ^225^Ac-labeled PSMA are reserved to PC patients after failure of all approved therapies, and generally a limited life expectancy is anticipated; this can affect the duration of possible side effects as well. Some authors have investigated the possible synergic therapeutic effect of ^225^Ac-PSMA after previous administration of ^177^Lu-PSMA. A recent meta-analysis highlights the possible development of cancer resistance as a drawback of a previous exposure to ^177^Lu [174]. Other authors tried a simultaneous administration of ^177^Lu-PSMA and ^225^Ac-PSMA in late-stage crPC patients to enhance the response rate [175,176].The PSMA determinant is physiologically expressed in the salivary glands by acinar and ductal cells. A risk of salivary gland toxicity in the case of administration of alpha-emitter radiolabeled PSMA is possible [177]. A risk-to-reward ratio should be evaluated to determine the eligibility criteria of PC patients, the isotope of choice, and the optimal therapy to be administered. Earlier α-therapy administration should be carried out in the context of prospective studies to reduce the treatment administered according to optimal therapeutic efficacy. Therapeutic window optimization will be a strong argument for a more controlled and systematic assessment of radioligand therapy.

## 7. Conclusions and Future Perspectives

Promising compounds labelling radionuclides are ready to deal with the limitations of the first released theragnostic radioligands. Salivary gland toxicity is a major challenge because it is frequent and can be irreversible. The PSMA uptake mechanism in the salivary glands is not clear, but some strategies to decrease accumulation are currently available. Nevertheless, long-term salivary gland toxicity needs to be investigated. A recent pilot study investigated the use of cold DCFPyL instillation into the salivary glands in order to decrease salivary uptake, mitigating xerostomia in patients scheduled for radioligand therapy [178]. Numerous studies have recently evaluated the application of Cu as PET/TC radiotracer in oncology because of its capacity to act as diagnostic and theragnostic radiotracer. It can produce β+ emissions and high-resolution PET images, while β and Auger electrons emission are suitable for targeted radiotherapy. The production of ^64^Cu is convenient: due to its long half-life, it can be produced by a single center and distributed to several PET centers, even if far away. Cu is essential for multiple biological functions, such as cellular respiration, redox reactions, cellular adhesion, and connective tissue synthesis. High serum Cu levels have been found in some tumors, and a correlation with disease stage has been demonstrated [179]. ^64^CuCl2 as a PET tracer in PC has been explored in few studies. Capasso et al. [180] compared ^64^CuCl2 PET/CT with multiparametric MR for staging purpose. The detection rate for primary tumors was similar, and the radiotracer had no urinary excretion and no side effects. Piccardo et al. [181] studied 50 patients with biochemical relapse with ^64^CuCl2 PET/CT, ^18^F-Choline PET/CT, and MR. They found that ^64^CuCl2 PET/CT was better than ^18^F-Choline PET/CT and multiparametric MR in metastatic detection. A high liver uptake was observed, but no organ-specific toxicity was detected. From a theragnostic point of view, the potential of ^64^CuCl2 in PC has only been evaluated in an in vitro setting. PSMA-directed molecular imaging is currently evolving through machine-learning approaches. Leung et al. described an automated deep-learning method comparing conventional and semi-automated thresholding-based methods for ^18^F-DCFPyL PET/CT evaluation in 207 patients [182]. They found that a deep-learning approach can help with more accurate segmentation and can lead to better therapy monitoring and future care planning. Machine learning and radiomics are emerging topics in clinical imaging. It can extract multiple features from pathological findings and potentially define new markers of disease [183,184,185]. Radiomics has the potential to further increase the value of imaging in PC management; nevertheless, its introduction into current clinical practice is full of questions, as emphasized by several radiomic studies [186,187,188,189]. Several approaches have been proposed and standardization is the major issue.

Recently, a new beta emitter, ^161^Tb (half-life = 6.89 days; Eβ͞av = 154 keV), has been proposed as a potential player in the radio-theragnostic playground in various types of cancer, including PC. Labeled with PSMA, it seems to have a better emission profile than ^177^Lu regarding adsorbed dose, because of its rich Auger electron emission rate [190,191]. Among alpha emitters, ^212^Pb and ^213^Bi with a short half-life (10.64 h and 1 h, respectively) [192], ^227^Th (half-life 18.7 days), and ^211^At (half-life 7.2 h) are promising radioisotopes. In particular, ^211^At provides a better biodistribution and strong labelling of PSMA, decreasing the dose delivered to non-targeted tissue, especially in regard to the salivary glands [193]. Furthermore, ^211^At production cost is considerably lower than other alpha emitters, resulting in a clear advantage for future application [194]. However, dosimetry remains the main challenge, requiring a robust body of investigation to optimize the potential of PSMA theragnostics.

## Figures and Tables

**Table 1:** Available tracers for PET/TC prostate cancer imaging.

Compound	Physiological Uptake	Target	Benefits	Drawbacks
^18^F-Choline and ^11^C-Choline	Liver, spleen, pancreas, kidneys, adrenal glands, salivary glands, bowel, and bone marrow	Choline kinase activity: upregulated in PC cells, especially metastatic cells, but also seen in other cancer cells	- Role in BCR, especially extraprostatic disease	- Renal excretion which may limit detection of disease in retroperitoneum and pelvis, especially with ^18^F-choline - Short half-life of ^11^C-choline limits it to in-site cyclotron production - Limited role in primary staging - PSA-dependent (>1.4 ng/mL) - ADT may impact detection of disease
^18^F-Fluciclovine	Liver, pancreas, lung, red bone marrow, and myocardium, and with increasing time there is uptake in skeletal muscle	Amino acid transporters: upregulated in PC but also expressed in a wide variety of cancers.	- No significant renal excretion - Short uptake time (4–10 min) - Role in BCR, especially extraprostatic disease	- Significant bone marrow uptake which may limit detection of bone metastases - ADT may impact detection of disease
^68^Ga-Prostate-Specific Membrane Antigen and ^18^F-Prostate-Specific Membrane Antigen	Kidneys, salivary glands, gastrointestinal tract, lacrimal, thyroid, adrenal, prostate glands, blood pool, vertebral bone marrow, and testes	PSMA: transmembrane glycoprotein with folate hydrolase activity, produced primarily in cell membranes of prostate epithelial cells with upregulation in PC	- Role in BCR and primary staging (in unfavorable intermediate and high-risk PC patients), superior to conventional imaging in detection of extraprostatic disease, especially nodes. - Superior detection of extraprostatic disease in BCR compared to choline and Fluciclovine PET - ADT improves the detection of disease	- Limited role in primary staging - PSA-dependent (>1.4 ng/mL) - Renal excretion, which may limit diagnosis of disease in retroperitoneum and pelvis - Cyclotron produced, which limits availability - PSA-dependent (>0.2 ng/mL)
^18^F-Pifluflolastat	Lacrimal glands, salivary glands, liver, spleen, small intestine, and kidneys	PSMA: folate hydrolase transmembrane glycoprotein, expressed primarily in cell membranes of prostate epithelial cells with upregulation in PC	- Role in BCR and primary staging (in unfavorable intermediate- and high-risk PC) - Extremely low renal excretion - ADT use may increase detection of disease - ^18^F is extensively available	- Significant liver uptake, which may lower the detection of metastatic disease but not common localization - PSA-dependent (>0.2 ng/mL)
^18^F-Fluorodeoxyglucose	Central nervous system, liver, spleen, kidneys, bladder, bowel	Takes advantage of the Warburg effect in cancer cells	- High sensitivity	- High urinary system uptake - Low specificity, cannot differentiate neoplastic or inflammatory uptake

ADT: Androgen Deprivation Therapy, BCR: Biochemical Cancer Recurrency, PSA: Prostate-Specific Antigen.

## Data Availability

The data presented in this study are available in this article.
